# Automated Signal Processing Applied to Volatile-Based Inspection of Greenhouse Crops

**DOI:** 10.3390/s100807122

**Published:** 2010-07-28

**Authors:** Roel Jansen, Jan Willem Hofstee, Harro Bouwmeester, Eldert van Henten

**Affiliations:** 1 Wageningen University, Farm Technology Group/P.O. Box 17, 6700 AA Wageningen, The Netherlands; E-Mail: JanWillem.Hofstee@wur.nl; 2 Wageningen University, Laboratory of Plant Physiology/P.O. Box 658, 6700 AR Wageningen, The Netherlands; E-Mail: Harro.Bouwmeester@wur.nl; 3 Wageningen UR Greenhouse Horticulture / P.O. Box 644, 6700 AP Wageningen, The Netherlands; E-Mail: Eldert.vanHenten@wur.nl

**Keywords:** automated, signal processing, plant volatiles, greenhouse

## Abstract

Gas chromatograph–mass spectrometers (GC-MS) have been used and shown utility for volatile-based inspection of greenhouse crops. However, a widely recognized difficulty associated with GC-MS application is the large and complex data generated by this instrument. As a consequence, experienced analysts are often required to process this data in order to determine the concentrations of the volatile organic compounds (VOCs) of interest. Manual processing is time-consuming, labour intensive and may be subject to errors due to fatigue. The objective of this study was to assess whether or not GC-MS data can also be automatically processed in order to determine the concentrations of crop health associated VOCs in a greenhouse. An experimental dataset that consisted of twelve data files was processed both manually and automatically to address this question. Manual processing was based on simple peak integration while the automatic processing relied on the algorithms implemented in the MetAlign™ software package. The results of automatic processing of the experimental dataset resulted in concentrations similar to that after manual processing. These results demonstrate that GC-MS data can be automatically processed in order to accurately determine the concentrations of crop health associated VOCs in a greenhouse. When processing GC-MS data automatically, noise reduction, alignment, baseline correction and normalisation are required.

## 1. Introduction

Costs associated with pests and diseases of greenhouse crops are high and likely to get much higher in the future. Reliable estimates of these costs are not available and greenhouse growers are notably reticent about reporting their losses [1]. Nonetheless, evidence for high costs is reflected in pest control expenditures. For instance, in the UK, the total cost of pest control in greenhouses (biological control agents, pesticides, monitoring and labour) was estimated at €8,500–18,000 per hectare per season (converted from UK currency) [2]. High costs provide an incentive to invest into costly and risky research and development of new technologies for detection of pest and disease threats at an early stage. An early detection would facilitate immediate action and prevent further spread by controlling the problem right at the source.

Plants release volatile organic compounds (VOCs) induced by the presence of pests and pathogens, [3–5]. Therefore, a novel approach to the detection of pests and pathogens might be based upon the analysis of air samples for the presence of these VOCs. Different types of instruments including electronic noses, biosensors, and gas chromatograph—mass spectrometers (GC-MS) have been used to analyse air for VOCs [6–8]. From a technological point of view, GC-MS is preferred because it shows a favourable combination of high selectivity and resolution, good accuracy and precision, wide dynamic concentration range, high sensitivity and the prospect for onsite application [9,10]. Unquestionably, GC-MS systems are expensive and costly to maintain. But, the price for GC-MS systems has dropped significantly and at the same time more robust GC-MS systems have been developed [11–13]. These developments leads one to expect that GC-MS might be used for the detection of pests and pathogens in greenhouses in the future.

A widely recognized difficulty associated with GC-MS application is the large and complex datasets generated by this instrument. As a consequence, experienced analysts are often required to process this data in order to determine the concentrations of the chemical compounds of interest [14]. Manual processing is time-consuming, labour intensive and may be subject to errors due to fatigue. These aspects are considered to be the limiting factors in the effective application of GC-MS based crop health monitoring in the 21st century. Developments in computer technology and software have increased the opportunity to automatically process GC-MS data within a reasonable time.

Numerous software packages (reviewed in [15]) have been developed for the automatic extraction of relevant information from complex GC-MS data. The algorithms implemented in these software packages rely on digital filters and univariate statistics for data smoothing, noise reduction, and baseline correction [16]. Additional alignment algorithms are often implemented to correct for chromatographic peak shifts [17]. The majority of these software packages have their roots in metabolomics: ‘the study of the unique chemical fingerprints that specific cellular processes leave behind’ [18]. Often, these software packages are successfully used to find novel compounds that explain differences between large series of mass spectrometric data [19].

It is still unknown whether these algorithms are also useful for automatic extraction of signals that represent crop health associated VOCs in order to determine their concentrations in samples of greenhouse air. Thus, the objective of this study was to resolve this issue. In this study, the processing algorithms implemented in the MetAlign™ software package were validated for that purpose.

## 2. Materials and Methods

### 2.1. Experimental Dataset

**Figure 1.**
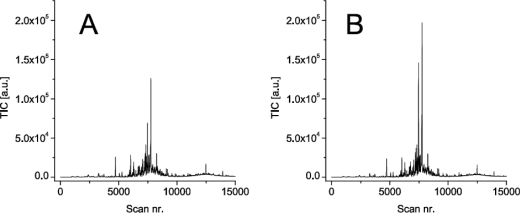
Typical chromatographic profiles obtained from analysing the air in a greenhouse. Data were obtained in week nr. 6; before (A), and directly after (B) damage of tomato plants (TIC = total ion current).

The experimental dataset employed in this study was acquired from the chemical analysis of air samples collected in a small-scale greenhouse. Throughout a six week growing period, the air inside this greenhouse was sampled directly before and just after artificial damage of a tomato crop. The artificial damage was imposed to the plants on a weekly interval and was supposed to simulate plant damage similar to that caused by plant health issues such as herbivore infestation or pathogen infection. The analysis of the resulting twelve air samples were performed offline using a gas chromatograph coupled to a mass spectrometer (GC-MS). The simplest data output from the mass spectrometer analyzer is a measurement of the total ion current strength (TIC) *versus* time. This is basically a chromatographic output representing a summation of the signal strength of all the ions produced by the mass spectrometer at a given time. Two typical examples of such chromatographic output obtained before and after damage of the tomato plants are presented in Figure 1.

**Figure 2.**
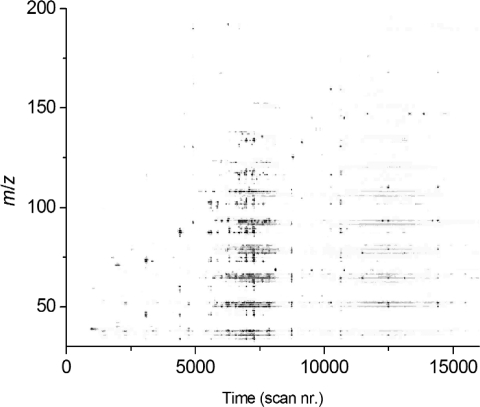
Three dimensional gas chromatography—mass spectrometry data display. Data were obtained in week nr. 6 before damage of tomato plants. Light grey colours represent low intensities of the corresponding *m/z* values while dark grey colours represent high intensities of the corresponding *m/z* values. (*m/z* = mass-to-charge ratio)

The actual data output content is much more complex since the data block produced is three dimensional; TIC *versus* time *versus* mass-to-charge ratios (*m*/*z*). More details can be found in McMaster [20]. A graphical way to present the three dimensional structure of GC-MS data is provided in Figure 2.

### 2.2. The Experimental Equipment and the Instrumental Settings

The air samples were collected by purging 18 L of air from the greenhouse through stainless steel cartridges (Markes International Ltd, Lantrisant, UK) packed with 200 mg of Tenax-TA 20/35 (Grace-Alltech, Breda, The Netherlands). Air was purged through these cartridges at 300 mL min^−1^ for 60 min. The air samples were transferred to the laboratory for analysis. Before analysis, the cartridges were dry-purged with helium at ambient temperature with a flow of 100 mL min^−1^ for 10 min to remove water. Analytes were desorbed from the cartridges using thermal desorption at 250 °C for 5 min under a flow of 30 mL min^−1^ of helium, and subsequently concentrated in an electronically-cooled focusing trap at −5 °C (UltrA-TD™ and Unity™; Markes International Ltd). Analytes were then transferred to the column by heating the cold trap to 250 °C at approximately 40 °C s^−1^. To prevent overloading of the analytical system, most samples were split prior to injection. Air samples obtained when plants were relatively small were analysed in splitless mode while samples obtained in case of large plants were analysed at split inlet modes between 1:6 and 1:24.

A gas chromatograph was used to separate the mixture of analytes (Trace GC UltrA™; Thermo Electron Corporation, Austin, TX, USA). The capillary column (Rtx-5 MS, 30 m × 0.25 mm internal diameter × 1 μm film thickness; Restek, Bellefonte, PA, USA) was held at the initial temperature of 40 °C for 3.5 min followed by a linear gradient of 10 °C min^−1^ to 280 °C and a hold of 2.5 min resulting in an overall runtime of almost 33 min. The carrier gas was nitrogen of 99.999% purity and the column flow was approximately 1 mL min^−1^.

The mass spectrometry was performed on a quadrupole mass spectrometer (Trace DSQ™; Thermo Electron Corporation). The mass scan range was set from 45 to 450 amu (atomic mass unit) at a scan rate of 5077 amu s^−1^ and the electron ionization energy was set at 70 eV. The response of the mass spectrometer was assumed to be linear up to 2 × 10^8^ ion counts per mass.

### 2.3. Manual Processing of Data

Manual processing of data was carried out by extracting the signals representing four VOCs: 2-carene, *α*-phellandrene, limonene, and *β*-phellandrene. Reference samples of these target VOCs were purchased (Fluka, Buchs, Switzerland) and subsequently injected into the GC-MS to determine their scan numbers (retention time). The corresponding peaks in the total ion chromatogram were manually located at these scan numbers. The TIC areas underneath these peaks were manually integrated using an appropriate software package (XCalibur 2.0; Thermo-Finnigan, San Jose, CA, USA). This software package was also used to extract the corresponding peak areas in the selective ion chromatograms (SIC) using *m/z* 93 as characteristic fragment. The ratio between the TIC areas and SIC areas, and results from a calibration were used to quantify VOC concentrations. The calibration procedure itself has been described before [8].

### 2.4. Automated Processing of Data

The GC-MS data was automatically processed by the MetAlign™ software package (version 040806) on a Pentium IV 1.5 GHz PC. The following steps were carried out: (1) data smoothing by digital filters related to the average peak width, (2) estimation and storage of local noise as a function of retention time and mass peaks, (3) baseline correction of mass peaks and introduction of a threshold to realise noise reduction, (4) scaling, calculation and storage of peak maximum amplitudes, (5) between chromatogram alignment, (6) iterative fine alignment by including an increasing number of mass peaks with lower signal-to-noise (S/N), significant difference filtering at user-defined significance thresholds and minimum x-fold ratios and (7) output of data back to the MS-platform. The algorithms implemented in the MetAlign™ software package have been disclosed and published in [21].

To correct for the split levels used, data were scaled to the chemical compound naphthalene (*m/z* = 128 at scan nr. 9520). Naphthalene was selected for scaling because this compound is not released from plants and was always present in almost constant concentration inside the greenhouse [22]. Scaling to naphthalene was also used to correct for variability in GC-MS sensitivity, e.g., due to contamination of the ion source. The quantification of VOC concentrations followed the procedure in Jansen *et al.* [8] corrected for MetAlign’s peak area to intensity transformation. Parameters of MetAlign were set according to the specific scaling requirements and the chromatographic and mass spectrometric conditions used in the experiments (Table 1).

## 3. Results

### 3.1. Data pre-processing with MetAlign

**Figure 3.**
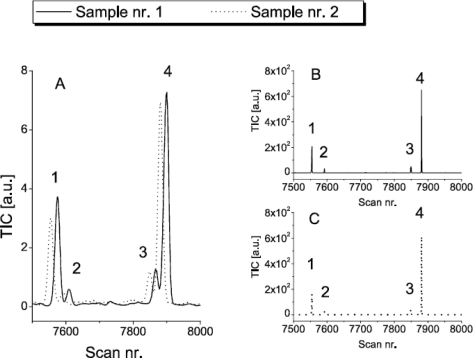
Impression of data pre-processing for signals that represent the concentration of (1) 2-carene, (2) *α*-phellandrene, (3) limonene, and (4) *β*-phellandrene. Provided are: (A) unprocessed data of sample nr. 1 and nr. 2; (B) baseline corrected, scaled, noise reduced and aligned data of sample nr. 1; (C) baseline corrected, scaled, noise reduced and aligned data of sample nr. 2. TIC = total ion current.

Two data files were randomly selected to illustrate the implemented pre-processing algorithms of MetAlign. These two data files showed significant difference in the scans numbers of the target VOCs (Figure 3). The phenomenon of drifted peaks and the effect of pre-processing the data are illustrated in Figure 3. This figure represents the effect of pre-processing the two data files in a small part of the chromatogram (scan nr. 7,500–8,000) and illustrates the effect of baseline correction, noise reduction, scaling and alignment.

**Figure 4.**
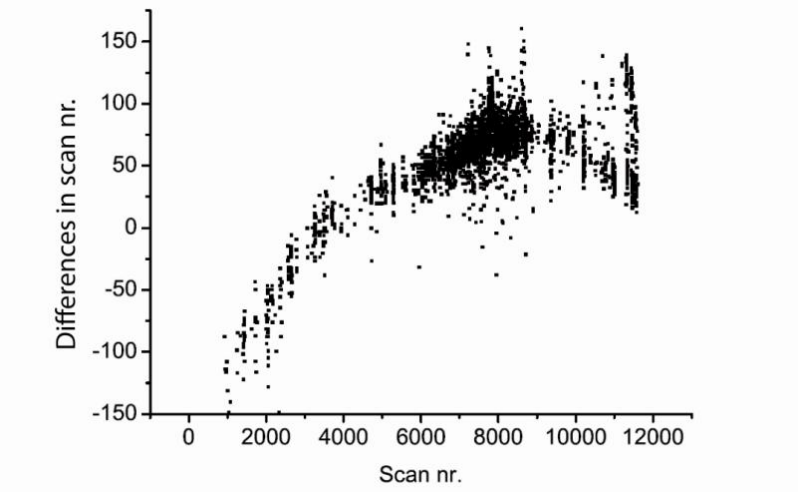
Differences in scan numbers for signals representing volatile organic compounds detected in gas chromatography—mass spectrometry analysis.

Figure 3 provides the signals representing the four target VOCs. Besides the differences in scan numbers of these VOCs, more than 3,000 signals showed differences in scan numbers (Figure 4). These differences in scans were especially large for high-volatile compounds which elute early (scan nr. <2,000) and for low-volatile compounds which elute late (scan nr. >8,000).

### 3.2. Manual Processing *Versus* Automated Processing: Detection and Concentration of VOCs

**Figure 5.**
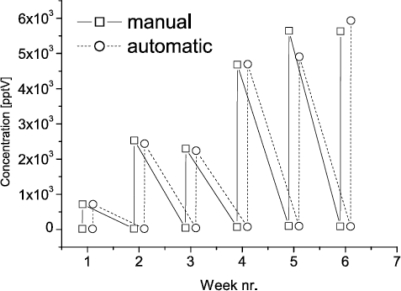
Time course of the concentration of 2-carene after manual and automatic processing of gas chromatography–mass spectrometry data. The data points have been offset to allow comparison.

The result of manual and automated analysis of the twelve samples collected in the greenhouse experiments is shown in Figure 5. It shows the time series of the concentration of 2-carene during the six consecutive weeks before and after artificial damage to the crop. Figure 5 clearly demonstrates the strong correlation between results obtained with manual and automated data processing. Also, Figure 5 shows a distinct positive trend in the concentration of the target VOCs upon artificial damage. Similar correlations and trends were found for *α*-phellandrene, limonene, and *β*-phellandrene.

### 3.4. Manual *vs*. Automated Processing: Time Needed for Analysis

Besides an accurate assessment, the data should also be processed within reasonable amount of time. About 1 h was needed to manually process the twelve data files used in this study. The overall time needed to process the dataset automatically was approximately 10 min.

## 4. Discussion

The results of this study demonstrate that GC-MS data can be automatically processed in order to accurately determine the concentrations of crop health associated VOCs in a greenhouse. The processing of data was performed using MetAlign; a freeware software tool that has been effectively applied to process mass spectrometric data obtained from the quality control of fruits, plant-oil, drinking water, and grass [23,24]. This tool was also applied in the field of metabolomics which aims to develop and apply strategies for the global analysis of metabolites in cells, tissues and fluids [25]. This study demonstrates how knowledge obtained from that rapidly expanding field can be used in an agricultural engineering setting.

An important disadvantage of MetAlign is that the code is not open access, which hampers the implementation and prevents incorporation of new algorithms developed by researchers. This disadvantage could be overcome by the use of publicly available code, such as the Matlab code (The MathWorks, Natick, MA, USA) provided in [26].

Variation in sample size for different GC-MS analysis are difficult to avoid. Also the experimental dataset used in this study indicated a variation in sample size injected onto the GC column. The variation was derived from the differences in intensity of the peak corresponding to the naphthalene standard (not shown). This variation in sample size emphasizes the necessity for normalisation of data. MetAlign allowed the normalisation to one specific mass fragment. But, this procedure does not allow the selection of more fragments which was desirable in our case since similar fragments were located at similar retention times (not shown). In addition, it can be seen from the chromatographic profiles that there is need for baseline correction (Figure 1 and Figure 3A). The baseline correction algorithm performed by MetAlign turned out to produce an acceptable result (Figure 3B). This seems important as baseline correction is imperative for the automatic pre-processing of chromatographic data [27].

The observed variation in scan numbers of signals points to the presence of unwanted peak drifts between samples (Figures 3 and 4). From the literature it is known that small peak drifts are common in chromatographic data. These drifts are known to chromatographers and are due to changes in the columns during use, minor changes in mobile phase composition, drift in the instrument or interactions between analytes [28]. Peak drifts in the order of 1–250 scans were observed in our data. Peak drift was especially large for high-volatile and low-volatile compounds. This result suggests that the volatility of VOCs plays an important role in the automatic processing of GC-MS data.

GC-MS data should be corrected for peak drifts to accurately determine the concentration of VOCs in an automated fashion. Several algorithms are described in literature for the alignment of chromatographic data. The alignment procedure, also referred as peak matching, can be done with COWtool software [28]. This method relies on piecewise linear correlation optimised warping (COW). A second commonly used alignment algorithm is based on dynamic time warping (DTW). Tomasi *et al.* [29] studied these two different algorithms –COW and DTW- as pre-processing steps for chromatographic data. They concluded that time alignment corrections should be handled with great care and pointed to difficulties with respect to the judgement of performance. We also experienced difficulties to assess the result of an alignment as produced by MetAlign. It seems that a generally accepted benchmark method is lacking. Lin *et al.* [30] determined whether the inconsistency was due to amplitude differences or phase variations using a “lobster plot”. This graphical evaluation of the result of aligning might also be applied to our data. However, it should be kept in mind that this procedure becomes time consuming and more subjective when the number of samples increases.

Besides technical aspects, also economic aspects should be considered when comparing manual with automatic processing of GC-MS data. For routine GC-MS analysis, excluding data analysis, our laboratory charges 25 euro per sample. For this study, approximately 5 min per sample was needed for manually processing. Manual processing would then result in 20% extra costs at a labour cost of 60 euro per hour. An automatic procedure would thus result in significant cost reduction especially in case the number of target VOCs would increase, resulting in more time needed per sample.

Automatic processing would especially result in a cost reduction when large amounts of samples need to be processed, for instance when air samples from different locations within the greenhouse should be analysed. This could be achieved by the use of a multi-valve system connected to multiple tubes distributed across the greenhouse. These tubes allow transfer of air samples from distant sites to a central GC-MS. The spatial resolution in which samples are collected is then probably limited by the time needed to pre-concentrate the VOCs of interest in order to achieve the detection limits of the GC-MS.

Automatic data processing would reduce the costs of GC-MS application and extents its use to other agricultural applications such as quality control. Potato-tubers are among the agricultural products that could be checked for quality-loss based on the analysis of emitted VOCs [31,32]. Recently, this method was successfully applied at laboratory scale to monitor quality aspects of several other agricultural products including milk, meat, vegetables, grains, and fruits [33–36]. For such applications, automatic data processing is valuable but GC-MS instruments also need to become more robust and less expensive before they can be applied in an agricultural setting.

## 5. Conclusions

This research is a response to the need of automatic data processing for GC-MS-based crop health monitoring. The results of automatic processing of the experimental dataset resulted in concentrations similar to that after manual processing. These results demonstrate that GC-MS data can be automatically processed in order to accurately determine the concentrations of crop health associated VOCs in a greenhouse. When processing GC-MS data automatically, noise reduction, alignment, baseline correction and normalisation seem to be required. The automatic processing of GC-MS data would result in significant cost reduction, especially in the case where the number of target VOCs would increase, resulting in more time needed per sample.

## Figures and Tables

**Table 1.** MetAlign settings used to automatically process the experimental dataset.

**Setting**	**Value**
Retention begin (scan nr.)	0
Retention end (scan nr.)	15,000
Maximum amplitude	200,000,000
Peak slope factor	0.5
Peak threshold factor	1
Average peak width at half height	20
Scaling	Marker peak
Nominal mass	128 at scan nr. 9520
Initial peak search criteria : maximum shift begin of 1^st^ region	15
Initial peak search criteria : maximum shift end of 1^st^ region	50
Maximum shift per 100 scans	35
Pre-align processing	Iterative
Minimum S/N ratio	10
